# Unique and Conserved Features of the Barley Root Meristem

**DOI:** 10.3389/fpls.2017.01240

**Published:** 2017-07-21

**Authors:** Gwendolyn K. Kirschner, Yvonne Stahl, Maria Von Korff, Rüdiger Simon

**Affiliations:** ^1^Institute for Developmental Genetics, Heinrich Heine University Düsseldorf, Germany; ^2^Institute for Plant Genetics, Heinrich Heine University Düsseldorf, Germany; ^3^Cluster of Excellence on Plant Sciences (CEPLAS), Heinrich Heine University Düsseldorf, Germany; ^4^Department of Plant Breeding and Genetics, Max Planck Institute for Plant Breeding Research Cologne, Germany

**Keywords:** root meristem, stem cell niche, CLE peptide signaling, root architecture, root development, barley

## Abstract

Plant root growth is enabled by root meristems that harbor the stem cell niches as a source of progenitors for the different root tissues. Understanding the root development of diverse plant species is important to be able to control root growth in order to gain better performances of crop plants. In this study, we analyzed the root meristem of the fourth most abundant crop plant, barley (*Hordeum vulgare*). Cell division studies revealed that the barley stem cell niche comprises a Quiescent Center (QC) of around 30 cells with low mitotic activity. The surrounding stem cells contribute to root growth through the production of new cells that are displaced from the meristem, elongate and differentiate into specialized root tissues. The distal stem cells produce the root cap and lateral root cap cells, while cells lateral to the QC generate the epidermis, as it is typical for monocots. Endodermis and inner cortex are derived from one common initial lateral to the QC, while the outer cortex cell layers are derived from a distinct stem cell. In rice and *Arabidopsis*, meristem homeostasis is achieved through feedback signaling from differentiated cells involving peptides of the CLE family. Application of synthetic CLE40 orthologous peptide from barley promotes meristem cell differentiation, similar to rice and *Arabidopsis*. However, in contrast to *Arabidopsis*, the columella stem cells do not respond to the CLE40 peptide, indicating that distinct mechanisms control columella cell fate in monocot and dicot plants.

## Introduction

The root system of cereal crops from the family of *Poaceae* like barley, maize and rice is composed of different types of roots formed during consecutive developmental stages. In the embryo, the primary root primordium and the primordia of seminal roots are initiated (Luxová, 1986). The primary root is initiated below the scutellar node, while the seminal roots are formed later above the scutellar node from the mesocotyl. In the subsequent life of the plant, the largest part of the root system is built by shoot-borne post-embryonic nodal roots (reviewed in Orman-Ligeza et al., 2013). Primary root growth depends on cell division and expansion. Meristematic cells at the root tip are small and divide rapidly several times before they are displaced from the meristem. At the transition zone, they enter a phase in which they cease division and start to rapidly elongate and differentiate (elongation-differentiation zone) (reviewed in Ivanov and Dubrovsky, 2013). In *Arabidopsis thaliana*, the number of cells in the meristem increases after germination, until the meristem reaches its final size when the rates of cell division and the rate at which cells exit the meristem into the elongation-differentiation zone are balanced (Dello Ioio et al., 2007). The meristematic cells are derived from a group of stem cells located in the distal stem cell niche (Dolan et al., 1993). In many plant species, the stem cell niche is organized from a mitotically less active region termed the quiescent center (QC) (Clowes, 1984). In *Arabidopsis thaliana*, the QC is surrounded by a single layer of stem cells which give rise to the different tissues that make up the main body of the root (Dolan et al., 1993). The proximally located stem cells give rise to stele, endodermis and cortex, while the distal cells produce the root cap (columella), epidermis and lateral root cap cells. The QC maintains these stem cells in their undifferentiated state through short-range signaling (van den Berg et al., 1997). One of these signals depends on the homeobox transcription factor WUSCHEL-RELATED HOMEOBOX5 (WOX5) that is expressed in the QC and maintains both the distal stem cells and the size of the proximal meristem, together with other factors (Sarkar et al., 2007). The differentiating cells surrounding the stem cell niche, however, provide feedback-signals that serve to limit the size of the stem cell population. One of these is CLAVATA3/ENDOSPERM SURROUNDING REGION40 (CLE40), a peptide carrying a secretion signal and a conserved 14-amino-acid motif (CLE motif) near its C terminus (Cock and McCormick, 2001). CLE40 is expressed in the stele and in differentiated columella cells (Stahl et al., 2009). CLE40 signaling requires ARABIDOPSIS CRINKLY4, a receptor-like kinase that is expressed in the distal stem cells and in differentiated columella cells (De Smet et al., 2008; Stahl et al., 2009). CLE40 was shown to restrict *WOX5* expression in order to create a feedback regulation that maintains the size of the distal stem cell population (Stahl et al., 2009). A CLE peptide dependent pathway can also serve to promote premature differentiation of the proximal meristem, via an unknown pathway involving CLAVATA2 and CORYNE (Hobe et al., 2003; Fiers et al., 2005; Pallakies and Simon, 2014).

The basic structure of the meristem and the stem cell niche is generally similar between species like *Arabidopsis*, tomato, rice and maize. Their roots consist of the same cell types, which are the columella, lateral root cap, epidermis, cortex, endodermis, and stele. However, the number of cell files, their origin and the size of the stem cell domains differ between species. Firstly, the size of the QC varies considerably between species, ranging from four cells in *Arabidopsis* and rice to 400–900 in maize (Clowes, 1984; Dolan et al., 1993; Jiang et al., 2003; Ni et al., 2014). Secondly, maize and rice roots generate a larger number of cortex cell files than tomatoes (2-3 files) or *Arabidopsis* (1 file) (Lim et al., 2000; Rebouillat et al., 2009; Ron et al., 2013). In *Arabidopsis*, tomato and rice, both cortex and endodermis share an initial cell (Dolan et al., 1993; Ron et al., 2013; Ni et al., 2014). Epidermis and lateral root cap are derived from a common ancestor cell in *Arabidopsis*, whereas the epidermis initial of rice and maize is independent of the lateral root cap (Dolan et al., 1993; Clowes, 1994; Lim et al., 2000; Ni et al., 2014). To identify general mechanisms of root meristem development, more plant species from different evolutionary branches should be compared. Barley (*Hordeum vulgare*) is the fourth most abundant crop plant and of significant agronomic importance (FAO, 2014). The genome sequence of the barley cultivar (cv.) Morex was published in 2012 and serves as basis for molecular genetic studies (Mayer et al., 2012). While the adaptation of the shoot development to many different environments has been well studied, not much is known about the root development and the architecture of the barley root meristem was not previously described in detail (Luxová, 1986). Knowledge on the anatomy of the barley root provides the basis to understand root development and to study its molecular control mechanisms. Here, we have analyzed the root meristem of the barley cv. Morex at a cellular level, determined the size of the QC, the number and origin of the fundamental cell layers and cell division patterns in the stem cell niche. We further show that evolutionary conserved CLE-peptide dependent signaling pathways control meristem differentiation in the proximal meristem in barley, but in contrast to *Arabidopsis*, do not control the maintenance of columella stem cells.

## Materials and methods

### Plant growth

Seeds of the barley (*H. vulgare*) cv. Morex were husked and sterilized by washing the seeds in 70% EtOH shortly and in 5% sodium hypochlorite for 30 min on a shaker at 4°C. The seeds were then rinsed with autoclaved dH_2_O twice and plated on square plastic plates (120 × 120 mm) containing growth agar (0,5 g MES hydrate, 2,2 g Murashige & Skoog medium per liter, pH 5.7). Per plate, 5 seeds were placed 2 cm from the top of the plate into the agar. The plates were then stored for at least 2 days in darkness at 4°C for stratification and placed in a 16°C phytochamber with 24 h light at a 45° angle to the shelf for growth. *Arabidopsis thaliana* (Col-0) seeds were treated and grown as described in Stahl et al. (2009).

### Peptide treatment

The synthetic peptides were acquired from Thermo Fisher Scientific and Centic Biotec with the following amino acid sequences: HvCLE402p (MLOC_3686.1) REVPTGPDPIHH; AtCLE40p RQVHypTGSDPLHHK (Hyp = hydroxyproline); mCLE40p LPQHPHGRSDVT. The peptides were added to the growth medium at a final concentration of 1 μM and the seeds were grown on these plates as described above for 5 days after germination (DAG).

### RNA *in situ* hybridisations

Probes for the *HISTONE H4* (AK357536) mRNA were prepared from the whole coding sequence. The DNA was cloned into the pGGC000 entry vector of the GreenGate cloning system (Lampropoulos et al., 2013) and amplified including the T7 and SP6 promoter sites by PCR. RNA probes were produced as described in Hejátko et al. (2006). RNA *in situ* hybridisations were performed on roots of plants 8 DAG as described in Jackson (1991), except for the following changes: after fixing the tissue over night at 4°C in 4 % para-formaldehyde, 0.1% tween-20, 0.1% triton-x-100 in PBS, a Leica ASP 300 tissue processor was used for embedding with the following protocol: 1 h 50% Ethanol (EtOH), 1 h 70% EtOH, 1 h 95% EtOH plus Eosin Y, 1 h 100% EtOH plus Eosin Y, 1 h 100% EtOH, 1 h 100% EtOH, three times 1 h 100% Xylol, 20 min paraplast at 60°C, 10 min paraplast at 60°C. 10 μm sections were made at the microtome.

### Staining and microscopy

Modified pseudo-Schiff propidium iodide (mPS-PI) staining was performed as described for floral stalks in Truernit et al. (2008) on root tips of plants 8 DAG. The staining with Schiff reagent and PI was carried out using vacuum. The samples were examined with either a 25x oil objective with a numeric aperture (NA) of 0.8 using a Zeiss laser scanning microscope (LSM) 510 Meta or a 40x water objective with a NA of 1.20 using a Zeiss LSM 780. PI was excited with a 561 nm Argon laser with emission detection at 566–718 nm.

For cross sections of the root hair zone, roots were embedded in melted 5% agarose and sectioned manually with a sharp razor blade. Endodermis staining with berberine hemisulfate was carried out as described in Lux et al. (2005). The samples were examined with a 40x water objective with a NA of 1.20 using a Zeiss LSM 780. Green fluorescence was excited with a 488 nm Argon laser with emission detection at 490–544 nm. Transmitted light pictures were taken with a transmitted light detector (T-PMT).

EdU staining was performed with the Click-iT EdU Imaging Kit (Invitrogen) and the fluorophor Alexa568 as described in the manufacturer's manual with the following modifications: root tips of plants 8 DAG were covered with 10 μM EdU in dH_2_O and placed in the phytochamber for the respective incubation time. Root tips were fixed for 1 h under vacuum and permeabilized for 1 h at room temperature. The Click-iT reaction was carried out for 1 h under vacuum in darkness. DNA-counterstaining was performed with 1 μg/ml DAPI in PBS for 1 h in darkness under vacuum. The samples were cleared for around 14 days at 4°C in clearing solution described in Warner et al. (2014). The roots were examined with a 40x water objective with a NA of 1.20 using the Zeiss LSM 780. DAPI was excited with a 405 nm Diode with emission detection at 410–560 nm, Alexa568 was excited with a 514 nm Argon laser and emission was detected at 545–697 nm in a separate track. The pinhole was set to 2,05 Airy units. Pictures were taken with the tile scan function with 10% overlap, a threshold of 0.70 and automatically stitched by the microscope software.

RNA *in situ* hybridizations were examined with a plan-neofluar 20x objective with a NA of 0.50 or a plan-neofluar 40x objective with a NA of 0.75 using a Zeiss Axioskop light microscope.

### Data analysis

For the alignment of the CLE-motifs, the 12 amino acid CLE-motif of *Arabidopsis* CLE40 (Hobe et al., 2003) was used for a BLAST search of barley CLE homologs. Barley homologs were obtained from http://webblast.ipk-gatersleben.de/barley/ using the default BLAST parameter settings among high and low-confidence genes (Mayer et al., 2012). Alignments were performed using MEGA7 (Molecular Evolutionary Genetics Analysis version 7.0) for bigger datasets and a MUSCLE alignment.

Measurements of the meristem length, counting of the distal stem cells and segmentation of the z-stacks were carried out in Fiji (Schindelin et al., 2012). For meristem length measurements, the border between meristem and elongation zone was defined by the first cell in the outermost cortex cell layer that doubled in cell length compared to its distal neighbor and analysis was carried out qualitatively from direct observation (as described in Dello Ioio et al., 2007). Segmentation of cells in the z-stack of the stem cell niche was performed with the MorphoLibJ plugin and morphological segmentation (Legland et al., 2016). All plots were created in R (R Core Team, 2015). Statistical significances of the meristem length and the distal stem cell differences were determined by a two-tailed Student's *T*-Test with the indicated *p*-value. For image compilation, Adobe Photoshop was used. In the microscope images of the meristems contrast and brightness were changed in parts of the images, as the images were composed of single microscope images.

## Results

### Adapted mPS-PI staining allows whole mount imaging of barley root meristems and reveals that the barley meristem approximates a steady size within 6 DAG

**Figure 1:**
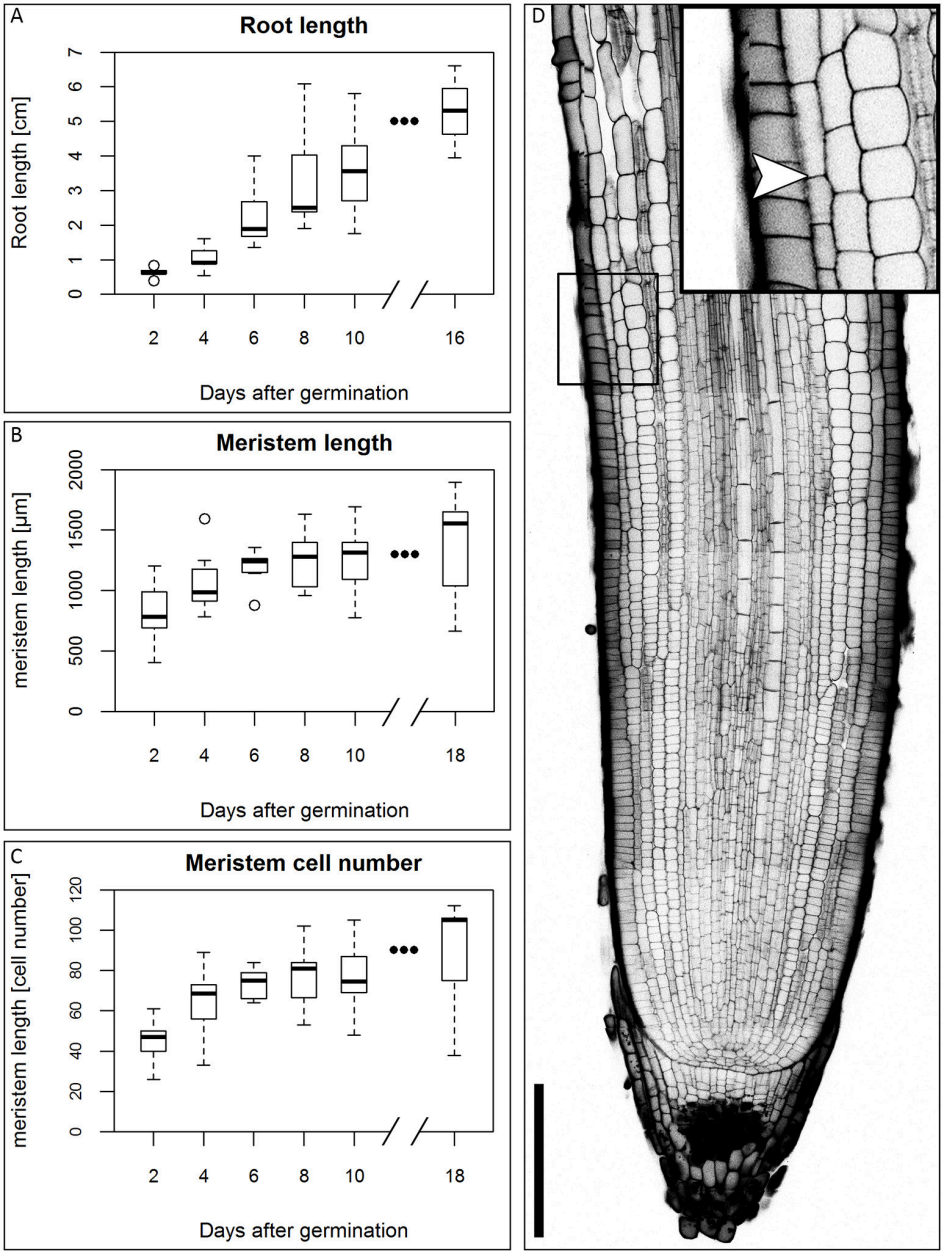
Growth of the barley root and the root meristem. **(A)** Root length of the barley root system at the first DAG; the average length of all roots of single plants was measured, *n* = 3–11 plants per time point, experiment was performed twice. **(B)** Meristem length of barley roots at different DAG in μm; the meristem growth is highest in the first DAG; after 6 DAG most of the meristems approximate a steady size; *n* = 9–17 per time point, experiments were performed twice. **(C)** Meristem cell number at different DAG; also the cell number of the meristem has reached a steady size after 6 DAG; n = 9–17 per time point, experiments were performed twice. **(D)** Representative picture of the root tip 10 DAG, starch and cell walls stained with mPS-PI staining; arrowhead indicates the transition zone; inset shows a magnification of the transition zone; scale bar represents 200 μm.

In the first days of seedling development, the seminal roots are the main root type involved in water uptake (Knipfer and Fricke, 2011), so they are of particular importance for development of the plant in water stress conditions. We therefore focused our study on this root type. There is no consensus in the literature on the number of seminal roots and the appearance of a primary root in different barley cultivars (Hackett, 1968; Luxová, 1986). Under our experimental conditions, the root system of the barley cv. Morex consists of 2–6 seminal roots that arise during the course of the first five DAG. Around 10 DAG, adventitious roots arise from the shoot. This is in accordance with the data of Knipfer and Fricke (2011) and Hackett (1969). Luxová (1986) distinguished primary and seminal roots of barley by position in the embryo, however, this was not possible for the cv. Morex without dissecting the embryo. Because we could not detect a phenotypical difference between any of the first roots, we made no distinction between primary and seminal roots. Within the first 16 DAG, the roots grew to an average length of approximately 5 cm in our growth conditions (Figure 1A). After germination, the number of cells in the meristem increases, until the meristem reaches its full size when the rates of cell division and cell elongation are balanced. However, problems in visualizing meristem cells in barley result from the thickness and size of the roots which make microscopy impossible without clearing. By adapting the technique of mPS-PI staining (Truernit et al., 2008) which was previously described for Arabidopsis flower stalks to barley, we could stain the root cell walls and starch granules in the root tip for confocal microscopy without previous sectioning. To measure the meristem size, we analyzed the outermost layer of the cortex cells next to the epidermis and defined the transition zone between the meristem and the elongation zone as prior exemplified for *Arabidopsis*. There, the transition between the meristem and the elongation zone is defined as the region where the first cortical cell doubles in size compared to its distal neighbor (Dello Ioio et al., 2007). The observation that the different seminal roots of a single barley seedling emerge over the course of up to 5 days following germination complicates this type of analysis, as the measured roots will differ slightly in age and, accordingly, developmental stage. This is reflected in the variation in root meristem lengths at each time point (Figures 1B,C). The meristem continued to grow, but growth slowed down approximately 6 DAG, while root length steadily increased in the monitored time window (Figures 1A–C).

### Cell layers in the barley root

**Figure 2:**
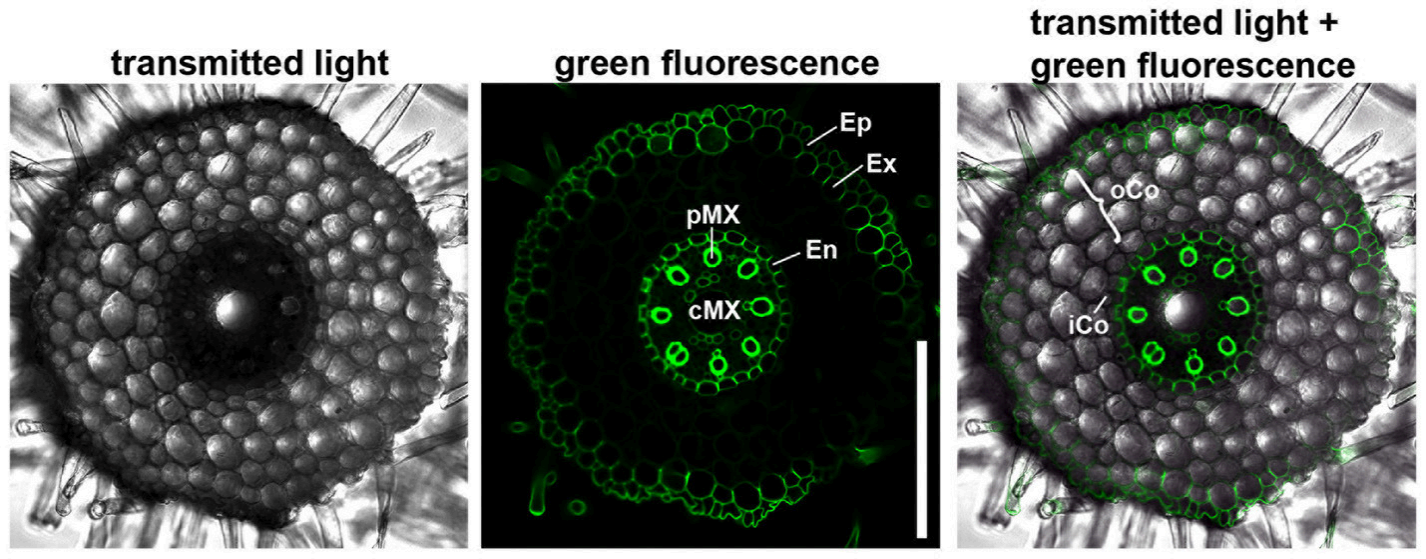
Anatomy of the barley root. Cross section of a root 8 DAG in the root hair zone, suberin stained with berberine hemisulfate; epidermis (Ep), exodermis (Ex), and endodermis (En) have suberized cell walls, while inner (iCo) andouter cortex (oCo) cells except for exodermis have not; eight peripheral meta xylem (pMX) vessels (autofluorescent) surround one central meta xylem (cMX) vessel in the central cylinder; scale bar represents 200 μm; *n* = 16, experiment performed three times.

We first characterized the number and identity of root cell files. Because cells are not yet differentiated in the root meristem, they cannot be distinguished only on the basis of their morphology. We therefore stained the suberized tissue in a cross section of the differentiated part of the root (root hair zone) with the fluorescent dye berberine hemisulfate (Figure 2). In this region of the root, the endodermis, the exodermis and the epidermis have suberized cell walls as diffusion barriers (Nawrath et al., 2013). We found that the barley root consists of one layer of epidermis, one layer of exodermis derived from the cortex, four layers of cortical cells and one endodermal layer. The cortex cells can be categorized into inner cortex (small cells) and outer cortex (larger cells) on the basis of their morphology. The central cylinder of the barley cv. Morex root comprises one large central and eight smaller peripheral metaxylem vessels, confirming similar observations on seminal roots of the cv. Golf by Knipfer and colleagues (Knipfer and Fricke, 2011). As the exodermis only forms in the differentiating zone of the root in a layer of cortical cells beneath the epidermis, it can be considered as cortex cell file in the root meristem (Enstone et al., 2003).

### The barley QC consists of around 30 cells

**Figure 3:**
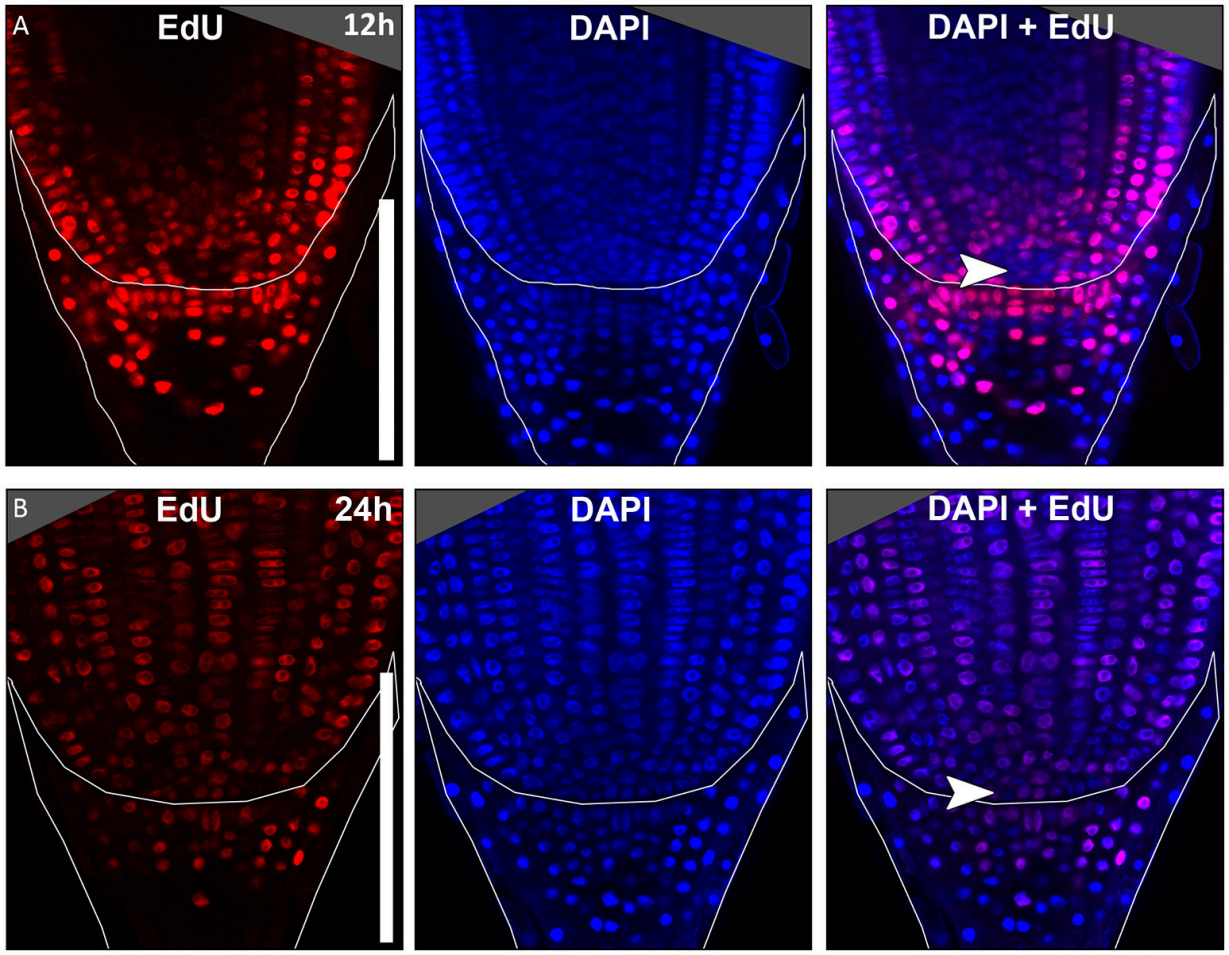
Cell division in the barley root tip. Roots of plants 8 DAG were treated with the cell division marker EdU (red), nuclei counterstained with DAPI (blue), merge image shows an overlay of both stainings. **(A)** Exemplary barley root tip after 12 h treatment with EdU. **(B)** Exemplary barley root tip after 24 h treatment with EdU; no area of low cell division rate is visible; scale bar represents 200 μm; arrow heads point to the putative QC region.

**Figure 4:**
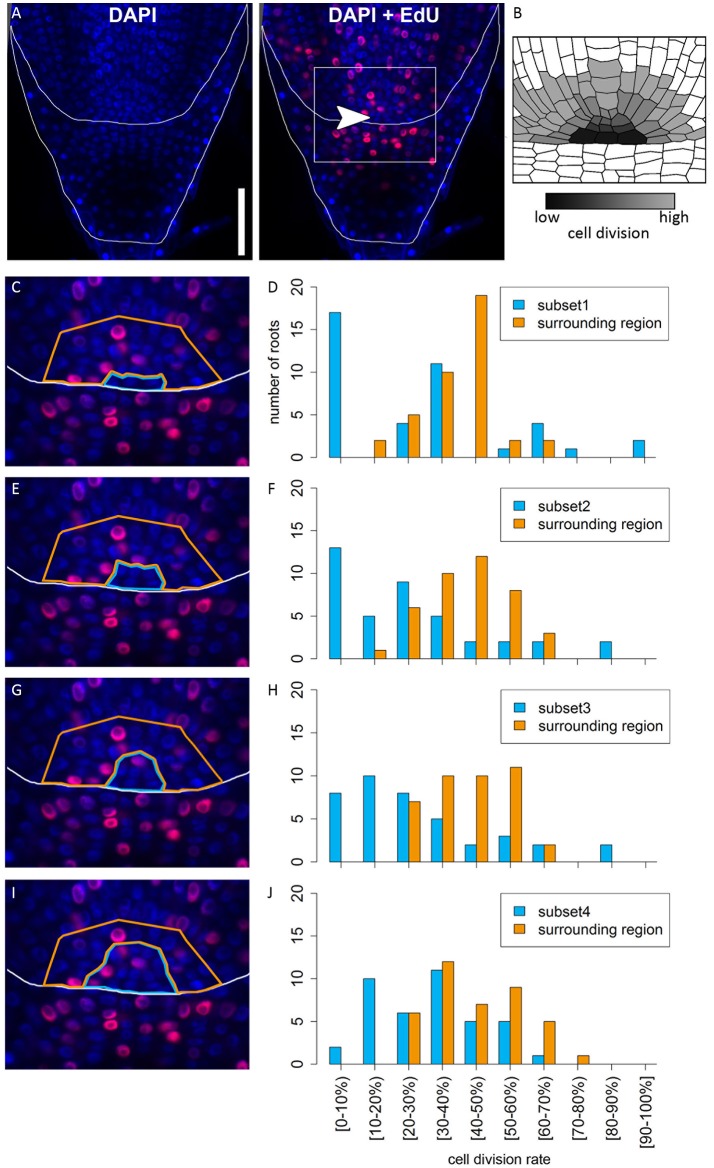
Analysis of cell division rates in different regions of the barley root tip. Roots of plants 8 DAG were treated with the cell division marker EdU (red) for 6 h and counterstained with DAPI as in Figure 3. **(A)** Exemplary barley root tip after treatment with EdU; merge image shows an overlay of DAPI and EdU staining; white arrow head points to the putative QC region; scale bar represents 100 μm. **(B)** Schematic view on the stem cell niche with cell division rates; cells with low division activity are marked with dark gray, cells with high division activity with light gray. **(C,E,G,I)** Magnification of the region marked in **(A)**; schematic view of the respective analyzed regions; bright blue marks the putative QC region in the center, orange marks the surrounding reference region. **(D,F,H,J)** Diagrams show the number of roots that showed cell division at the rate given on the x-axis in the respective area in the stem cell niche; different areas (bright blue and orange) refer to the areas marked by colors in **(C,E,G,I)**; square brackets for the cell division rates indicate that the following number is included in the column, while round brackets indicate that the numbers are excluded. **(C)** The putative QC includes only the four most central cells of the stem cell niche next to the root cap border (subset 1). **(D)** A large difference in cell division rate can be found between the putative QC region and the surrounding cells. **(E)** The putative QC includes the region from **(C)** plus around four proximal cells (subset 2); here again, there is an obvious difference in cell division rate between the QC and the surrounding cells, indicating that this subset enclosures the QC region **(F)**. **(G)** The putative QC includes the region from **(E)** plus around four proximal cells (subset 3), meaning that the putative QC region here includes 9 cells proximal to the root cap border. **(H)** Again, there is a difference in cell division rates between the putative QC region and the surrounding cells, indicating this subset still enclosures the QC region. **(I)** The putative QC region consists of the region marked in **(G)** plus one additional cell layer surrounding it (subset 4); here, the difference in cell division rate does not appear **(J)**, meaning that this subset probably includes more than only the QC region.

**Figure 5:**
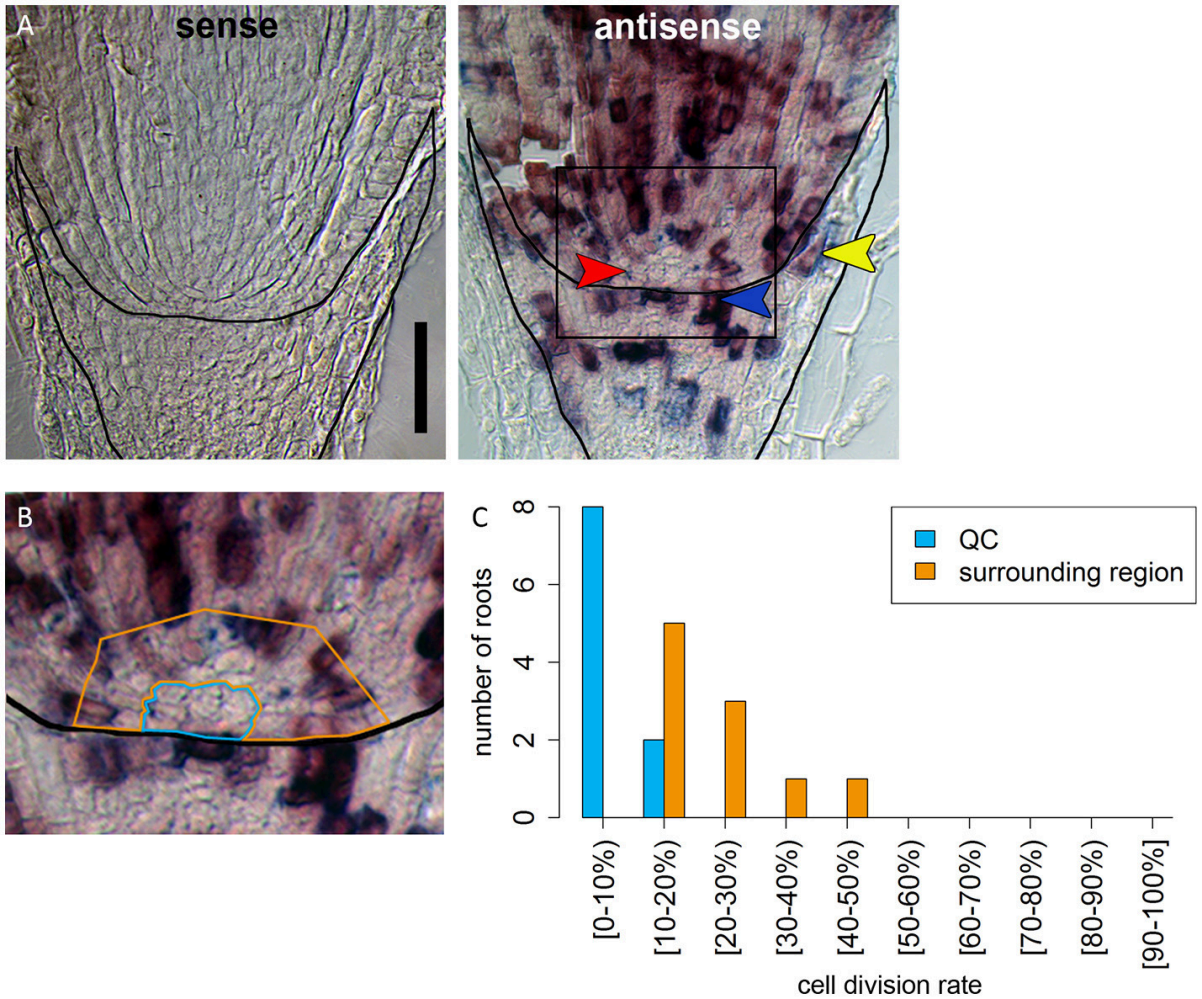
Identification of a region of slowly dividing cells in the barley stem cell niche. **(A)** RNA in *situ* hybridizations with probe against the S-phase marker *H4*; the sense control shows no staining; in the antisense sample, cells in the S-phase expressing *H4* are stained in purple; red arrow head in points to the putative QC region without *H4* expression; orange arrowhead points to cell divisions that form the lateral root cap; blue arrowhead points to cell divisions in the distal stem cells that build the columella; scale bar represents 100 μm. **(B)** Magnification of the region marked in **(A)**; orange and blue frame surround the regions analyzed in **(C)**; frame around the root marks the position of the root cap border. **(C)** Percentage of cells that have express *H4* as cell division marker in the respective area in the stem cell niche illustrated in **(B)**.

In many plant species analyzed, the QC serves to maintain adjacent stem cells in an undifferentiated state by short range signaling (Dolan et al., 1993; van den Berg et al., 1997; Ni et al., 2014; Kerk and Feldman, 1994). QC and stem cells of maize and *Arabidopsis* are characterized by their slower cell division rate in comparison to the surrounding cells (Clowes, 1984; Dolan et al., 1993). This quiescence could be necessary to protect the QC from DNA damage caused by DNA replication and allows to provide a pool of cells with an error-free genome for renewing the surrounding stem cells (Heyman et al., 2014). To investigate the cell division rate in the barley stem cell niche, we made use of two approaches, EdU staining and RNA *in situ* hybridization to detect expression of Histone H4, which is specifically expressed during S-phase. EdU is a thymidine analog that is incorporated into the DNA during DNA synthesis and hence labels cells in the S-phase of the cell-cycle (Kotogány et al., 2010). In *Arabidopsis*, a 24 h incubation with EdU labeled all cells in the stem cell niche except for the QC cells (Vanstraelen et al., 2009). In the barley root meristem, a 24 h treatment with EdU marked almost all nuclei in the stem cell niche (Figure 3B), while after a 12 h incubation with EdU 60 % of the roots (9/15) carried cells lacking EdU label in the putative stem cell niche (Figure 3A). Results of a 6 h EdU treatment were even more striking and 70% of all roots (30/44) lacked EdU incorporation at this position (Figure 4). Importantly, we could not detect any group of cells that was non-dividing over more than 24 h. To identify the QC cells, we analyzed the cell division rate in different subsets of the stem cell region in roots that were treated with EdU for 6 h (Figure 4). Notably, the cell division rates in the subsets revealed that a quiescence gradient in the QC region exists, with the highest quiescence, i.e. the lowest cell division rates in the cell layer adjacent to the root cap (subset 1) and increasing cell division rates in subsets 2, 3 and 4 (Figures 4B–I). Subset 3, with the most striking difference in cell division rate in comparison to the other subsets, is displayed in Figures 4F,G and includes around 9 cells in longitudinal sections (blue frame). The cell division rate in subset 3 ranged from 0 to 30%, while the cell division rate in the surrounding cells (orange region in Figure 4F) was predominantly in a range from 20 to 60% (Figure 4G). We therefore suggest that subset 3 cells represent the QC region. However, in 14/40 roots, more than 30% of the QC cells were EdU stained within a 6 h period (Figure 4G). To confirm this cell division pattern, we performed RNA *in situ* hybridisation with a probe detecting *Histone H4*. Cell division rates in subset 3 (blue frame in Figure 5B) ranged from 0–20 and10–50% in the surrounding region (orange frame in Figures 5B,C), supporting the previous identification of the QC in subset 3. Assuming that the QC has a hemispherical shape, we calculated that the entire QC consists of around 30 cells.

### Formation of epidermis, cortex, and endodermis

**Figure 6:**
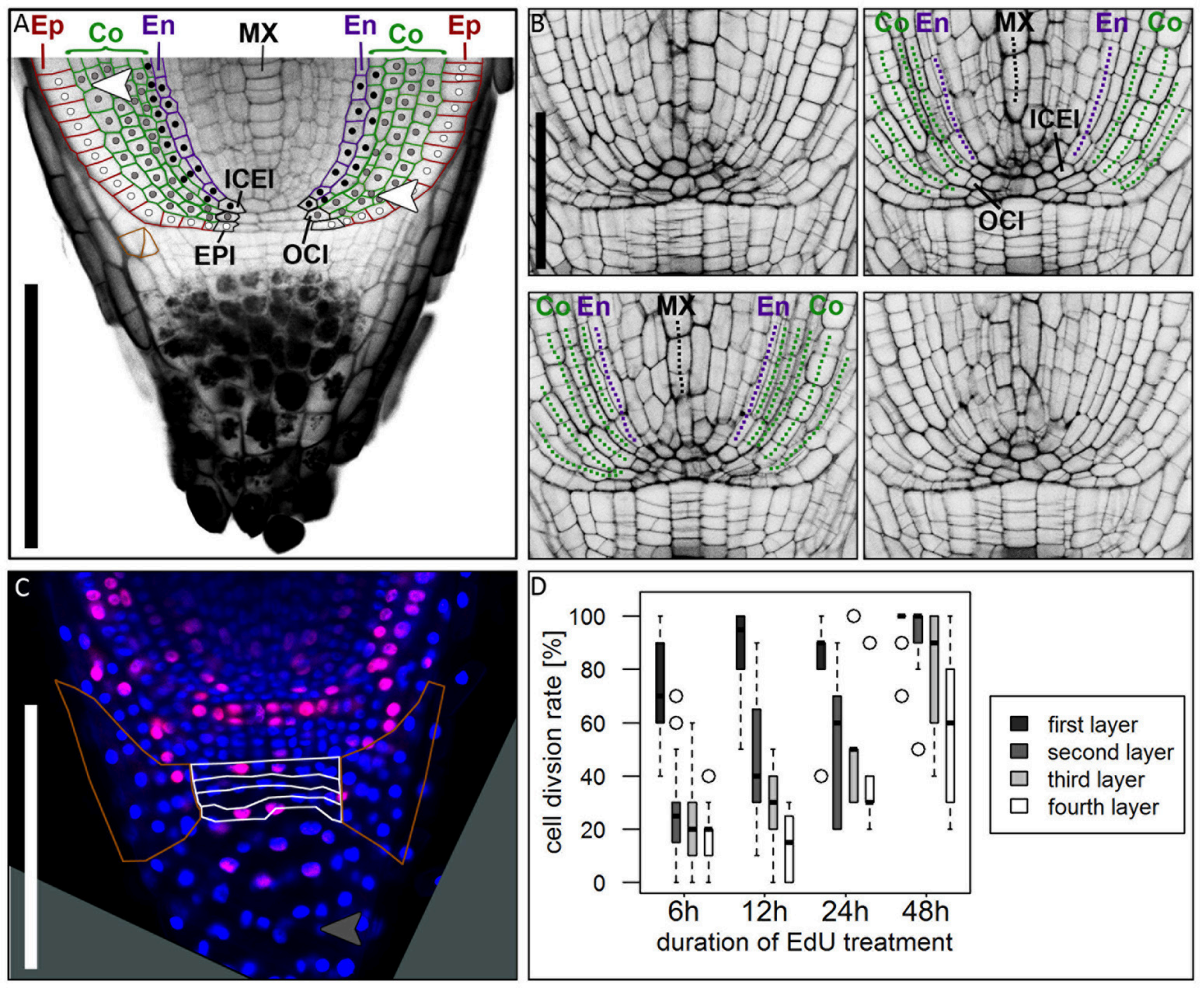
Cell division patterns in the root meristem. **(A)** Root tip stained with mPS-PI staining; colored cell walls mark the epidermal (red), cortical (green) and endodermal (magenta) cell files; white dots mark the cell lineage of the epidermis resulting from the arrangement of cell walls, derived from the epidermis inital (EPI); gray dots mark the cell lineage of the outer cortex cell layers, derived from the outer cortex initial (OCI); black dots mark the cell lineage of the inner cortex cell layers and the endodermis, derived from the inner cortex/endodermis initial (ICEI); white arrowhead points to a periclinal cell division in the cortex; orange cell walls marks an oblique cell division that forms the lateral root cap; Ep, epidermis; Co, cortex; En, endodermis; MX, metaxylem; scale bar represents 200 μm. **(B)** Longitudinal optical sections through the barley stem cell niche from the same z-stack presented in Supplementary Movie 1; Images were acquired at a distance of 5 μm; labels according to **(A)**, dotted lines follow the cell files in the respective colors; ICEI and OCI are visible in the center of the root, marked by appearance of the metaxylem and confirmed by tracing the cells in Supplementary Movie 1; scale bar represents 100 μm. **(C)** Root tip treated with EdU for 6 h; white borders mark the layers of undifferentiated cells distal to the root cap border, orange borders mark the lateral root cap; gray arrow head points to a cell division in the differentiated part of the root cap; root age 8 DAG; scale bars represent 200 μm. **(D)** Diagram showing the cell division rates of the layers of undifferentiated cells distal to the QC marked with white borders in **(B)** visualized by different EdU treatment times, 6, 12, 24, and 48 h (black = first layer distal to QC, dark-gray = second layer, light-gray = third layer, white = fourth layer); cell division rate is highest in the first layer distal to the QC, but increasing in the other layers after prolonged EdU treatment; *n* = 44 (6 h), 12 (12 h), 5 (24 h), and 14 (48 h); experiment was performed twice.

We studied mPS-PI stained barley roots to identify the clonal origin of the epidermal, the five cortical and the endodermal cell layers. By tracing the outlines of cells, we found that the endodermis and a variable number of cortex cell files share the same founder cell which locates adjacent to the QC (in 17/23 roots at 5–8 DAG). In most cases, a QC abutting inner cortex endodermis initial (ICEI) gave rise to the endodermal cell layer and the inner cortex cell layer, whose descendants remained distinctly smaller than the outer cortex cell layers (see Figure 2). However, in contrast to the model organism *Arabidopsis*, cell division patterns are less regular. We often found that cells at a distance to the QC underwent periclinal cell divisions, thereby generating additional cortex cell files (white arrow heads in Figure 6A). The outer cortex cell layers derived from a distinct outer cortex initial (OCI) that first generated two cortex cell files by alternating between anticlinal and periclinal divisions. Further periclinal division in descendants give rise to additional cortex layers. Serial optical sections confirm this cell division pattern (Figure 6B, Supplementary Movie 1). This pattern of cell layer generation differs from that in rice, where a ground tissue stem cell abutting the QC undergoes four rounds of asymmetric divisions to generate endodermis, cortex, exodermis and sclerenchyma cell layers (Rebouillat et al., 2009; Ni et al., 2014). In maize, the position and cell division pattern of stem cells has not yet been analyzed in detail (Hochholdinger et al., 2004a; Jiang and Feldman, 2005). In barley, the epidermis can be traced back to a dedicated stem cell adjacent to the QC (in 20/26 roots at 5–8 DAG) (Figure 6A). The origin of the vascular system and division patterns of vascular stem cells could not be traced back unequivocally by our mPS-PI staining method. Cell division patterns appeared highly variable, and reporter lines marking cell clones would be required to determine number and behavior of vascular initials. However, a single file of metaxylem cells is prominent in all roots, which can be identified at a distance of more than 5 cells from the QC (Figures 2, 6A).

### Formation and structure of the lateral root cap and the columella

At the root tip, we observed a distinct border that separates the starch-containing columella cells and the undifferentiated columella precursors from the proximal part of the root (Figure 6A). Differentiated columella cells contain starch grains that are necessary for gravitropism (Kiss et al., 1996). In rice, maize and *Arabidopsis* all cells of the columella and lateral root cap except for the columella stem cells contain starch grains (**Figure 10**; Dolan et al., 1993; Lim et al., 2000; Wang et al., 2014). In barley cv. Morex, we detected starch granules only in the five to six distal layers of the columella. The lateral root cap and on average four cell layers proximal to the differentiated root cap cells, however, lacked starch granules (Figures 1D, 6A, **9E**) and might act as columella stem cells. EdU treatment revealed that the first cell layer distal to the QC had a high cell division potential, which declined in the three more distal layers (Figures 6C,D). We occasionally also observed cell divisions in differentiated root cap cells (gray arrowhead in Figure 6C), which was also noted for columella cells in *Arabidopsis*, but not for rice (Wang et al., 2014; Hong et al., 2015). The lateral root cap originates from periclinal and oblique (in 6/30 roots at 5–8 DAG) cell divisions of lateral columella stem cells (Figure 6A, cells framed with orange), and lateral root cap cells maintain high division activity (marked by orange line in Figure 6C). Extended EdU staining over 48 h confirmed that most lateral root cap cells divided at least once within this time frame. *Histone H4* expression analysis supported the conclusions drawn from EdU stainings, showing that (1) all cells of the root cap remain division active, and (2) that the columella stem cell layer proximal to the QC maintains the highest divisional activity (blue arrow head in Figure 5A). The cell division pattern and cell wall arrangement at the position of the epidermis initial (EPI) indicates that the epidermal cell layer and the lateral root cap of barley are of independent origin, as it is typical for monocot roots (Figure 6A; Clowes, 2000; Rebouillat et al., 2009).

### The barley meristem is consumed upon CLE40 peptide treatment while the distal stem cells are unaffected

**Figure 7:**
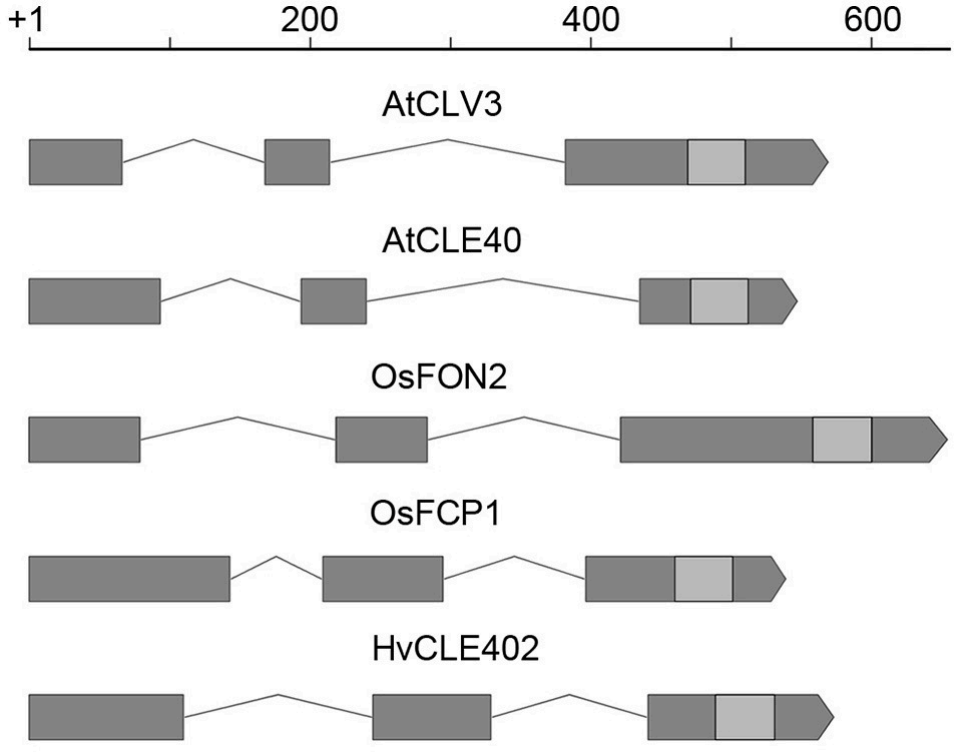
*CLE* gene structure in *Arabidopsis*, rice and barley. Gene structure of *CLV3, CLE40* (*Arabidopsis*) (Fletcher et al., 1999; Hobe et al., 2003), *FON2, FCP1* (rice) (Suzaki et al., 2006, 2008), and *HvCLE402* (barley); all of these *CLE* genes consist of three exons (gray boxes), two introns (lines connecting the boxes) and the sequence coding for the CLE-motif (light gray boxes); scale bar in bp.

**Figure 8:**
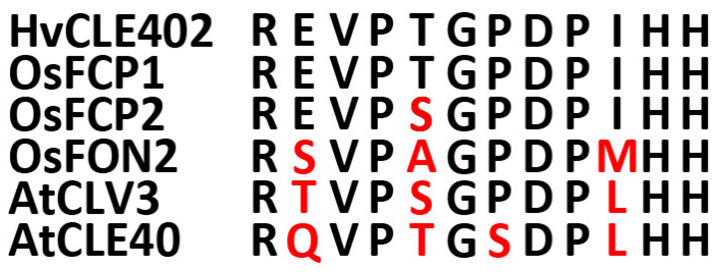
Sequences of the CLE-motifs of selected CLE genes from rice, *Arabidopsis* and barley. CLE-motifs of FCP1, FCP2, and FON2 from rice, CLV3 and CLE40 from *Arabidopsis* and CLE402 from barley (Suzaki et al., 2008). The CLE-motif from barley completely matches the one from FCP1.

**Figure 9:**
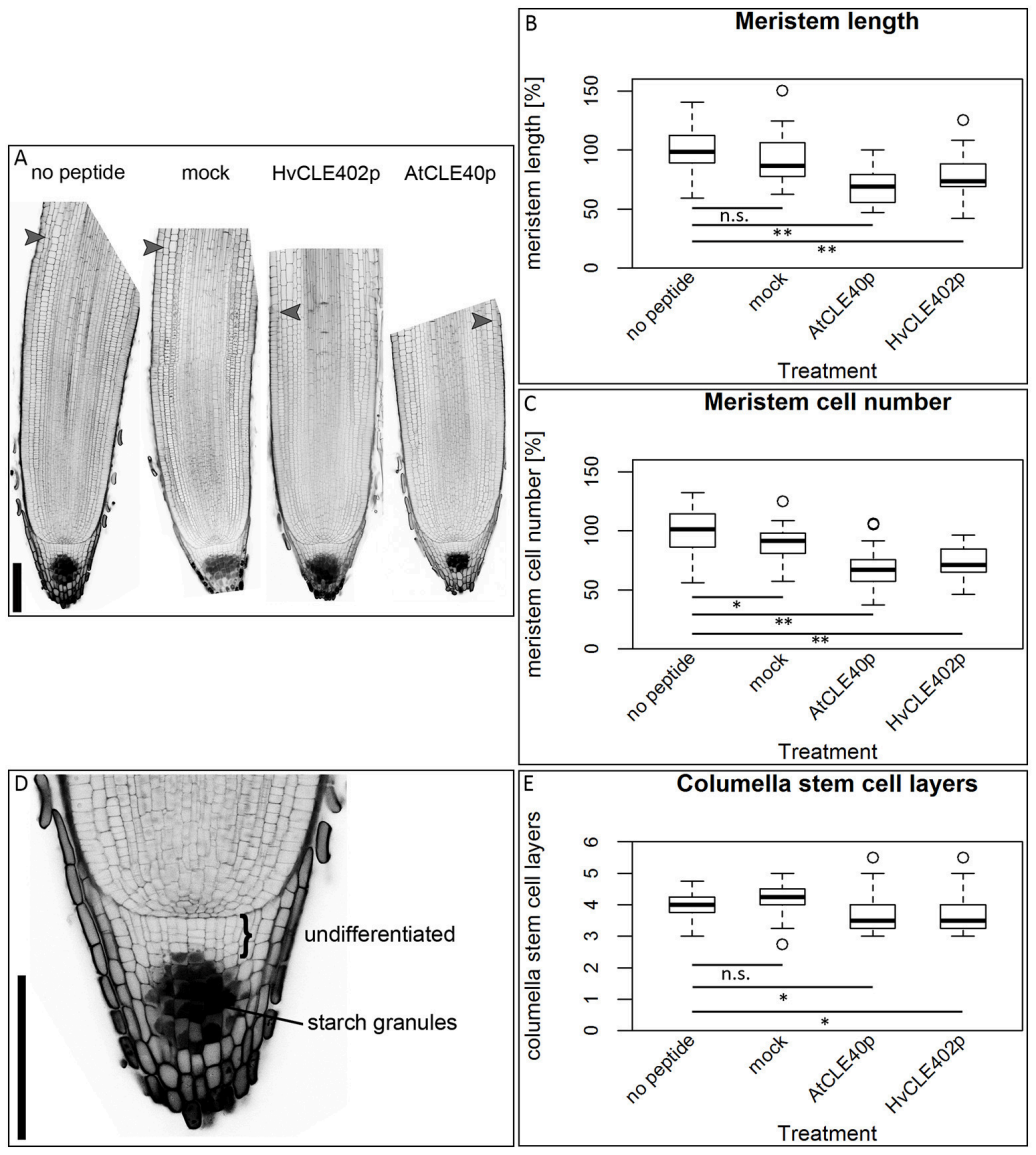
The barley root meristem and distal stem cell niche upon CLE40 peptide treatment. **(A)** The proximal meristem cells of the barley root differentiate prematurely when grown on medium containing either 1 μM HvCLE402p or 1 μM AtCLE40p compared to medium containing no peptide or 1 μM mock peptide (mCLE40p) for five DAG; arrowheads mark the transition zone between meristematic and elongation zone. **(B)** Meristem length measured in μm normalized to untreated plants (no peptide). **(C)** Meristem cell number normalized to untreated plants; asterisks indicate a significant difference between the respective treatments; n.s. = not significant; ^*^ = *p* < 0.05, ^**^ = *p* < 0.001; experiment performed 5 times; *n* = 17–54. **(D)** Exemplary distal root meristem with brackets marking the columella stem cells. **(E)** The number of distal stem cell layers is unaltered by peptide treatment; per root, four vertical columns were analyzed and the average is displayed in the diagram; experiments were performed 4 times; *n* = 14–31, scale bar 200 μm.

**Figure 10:**
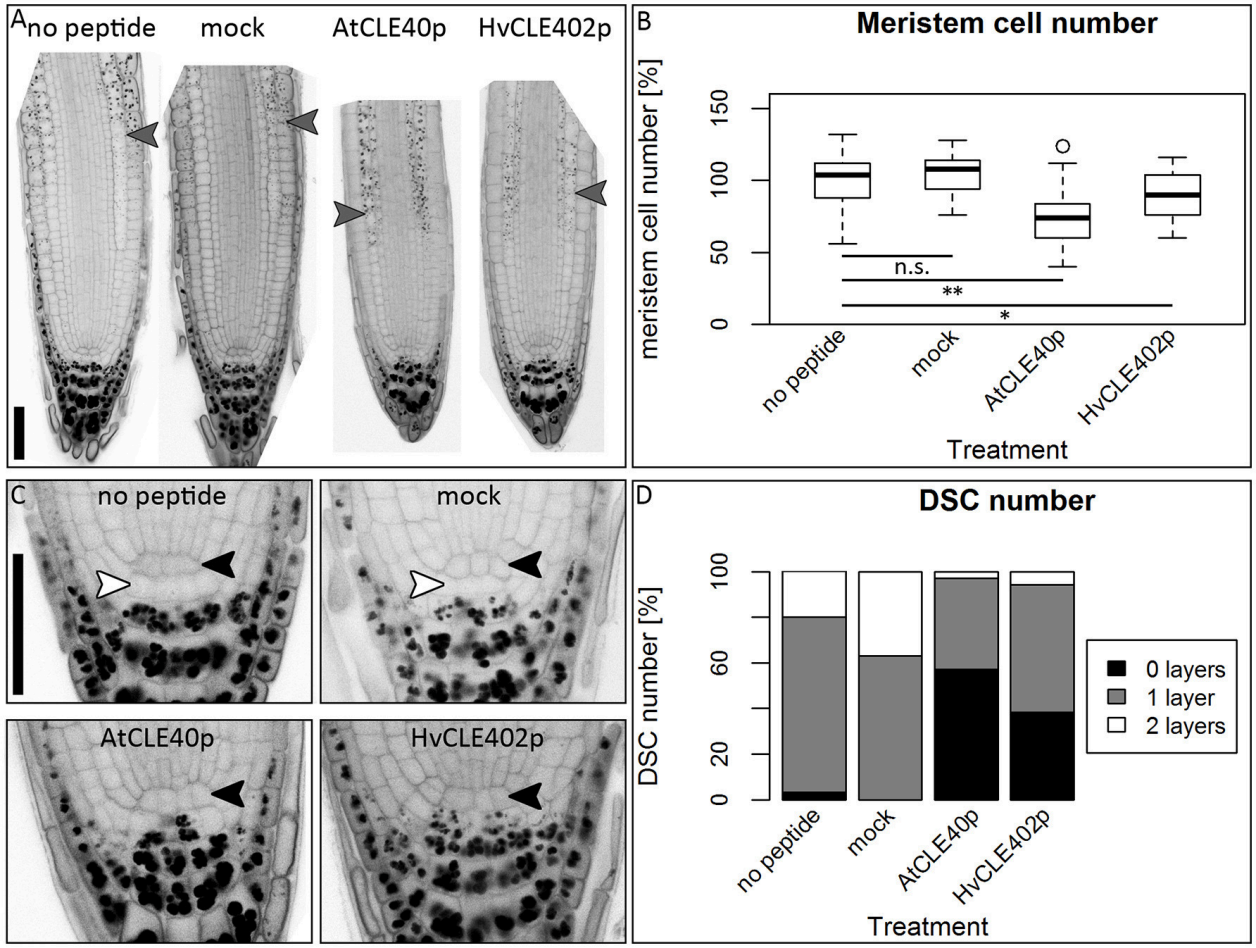
The *Arabidopsis* root meristem and the distal stem cell niche upon CLE40 peptide treatment. **(A)** Exemplary meristems of *Arabidopsis* roots upon 1 μM HvCLE402p, 1 μM AtCLE40p or mock treatment for five DAG; gray arrowheads mark the transition zone between meristematic and elongation zone. **(B)** In comparison to untreated or mock (mCLE40p) treated plants, growth on AtCLE40p or HvCLE402p leads to a reduced meristem size; meristem size was counted in cell number and normalized to untreated (no peptide) plants; asterisks indicate a significant difference between the respective treatments; n.s. = not significant; ^*^ = *p* < 0.05, ^**^ = *p* < 0.001. **(C)** Exemplary pictures of *Arabidopsis* distal stem cell niches upon HvCLE402p, AtCLE40p or mock treatment; black arrow heads mark the QC position, white arrow heads mark distal stem cells without starch; treatment with both AtCLE40p and HvCLE402p leads to a differentiation of the distal stem cells, visible by accumulation of starch granules. **(D)** Percentage of roots with no undifferentiated distal stem cells (DSCs) (black bars), one layer (gray bars), or two layers (white layers); experiment performed once; *n* = 27–34; scale bars 50 μm.

In *Arabidopsis*, a constant population of columella stem cells is maintained through a negative feedback regulation, involving the differentiated columella cells and the QC. The QC promotes columella stem cell fate in adjacent cells due to a non-cell autonomous function of the mobile transcription factor WOX5 (Pi et al., 2015). Differentiated columella cells, which are the descendants of the columella stem cells, express the secreted peptide CLE40 which acts via receptor kinases to confine *WOX5* expression (Stahl et al., 2009, 2013). An excess of CLE40 causes a rapid differentiation of stem cells toward columella cell fate. Additionally, CLE40 also regulates the size of the proximal meristem: here, increased levels of CLE40 can induce stem cell differentiation and loss of meristem activity (Hobe et al., 2003; Fiers et al., 2005). We now asked if a similar mechanism is acting in barley, and identified 21 CLE family peptides encoded in the available barley genome (Mayer et al., 2012). In *Arabidopsis*, CLE peptides are involved in a variety of developmental processes, but only two of them, CLE40 and CLAVATA3 (CLV3), act in meristem maintenance pathways. In rice, FLORAL ORGAN NUMBER2 (FON2) and FON2-LIKE CLE PROTEIN1 (FCP1) encode highly homologous CLE peptides, and of all CLE genes analyzed, only FCP1, FON2, CLV3, and CLE40 carry two introns, reflecting a common evolutionary origin (Fletcher et al., 1999; Hobe et al., 2003; Chu et al., 2006; Suzaki et al., 2006, 2008). Our search for CLE genes in the barley genome uncovered MLOC_3686 (named now HvCLE402) (Mayer et al., 2012), which carries two introns and encodes a CLE peptide highly related to FCP1 (Figures 7, 8). Treating barley plants with 1 μM synthetic HvCLE402 peptide consisting of the 12 amino acid CLE motif (HvCLE402p) or the *Arabidopsis* AtCLE40 peptide led to a significantly reduced root meristem length in comparison to untreated or mock treated plants (Figures 9A,B). We found that the number of proximal meristem cells was severely reduced (Figure 9C), indicating that the reduction of meristem size is likely caused by premature differentiation of proximal meristematic cells. This is consistent with observations made for the response of rice or Brachypodium to increased CLE peptide levels (Kinoshita et al., 2007; Czyzewicz et al., 2015). In the distal root meristem, 4 layers of columella stem cells give rise to the starch granule containing columella cells. In *Arabidopsis*, columella stem cell fate and number is negatively feedback regulated by CLE40 peptide, which is generated by differentiated columella cells. We asked if a similar regulation takes place in barley, and counted columella stem cell layers in peptide treated and untreated roots. Treatment with high levels of HvCLE402 or AtCLE40 peptides caused a minor, albeit statistically significant reduction of stem cell number, indicating that regulation of stem cell fate cannot depend solely on HvCLE402 (Figures 9D,E). Furthermore, we found that the HvCLE402 peptide can trigger both differentiation of columella stem cells and the proximal meristem cells in *Arabidopsis*, like the AtCLE40 peptide (Figure 10, Stahl et al., 2009), indicating that the CLE40 pathway controlling proximal meristem maintenance is highly conserved between monocots and dicots, while the distal meristem is only partially regulated through a CLE40 pathway.

## Discussion

**Figure 11:**
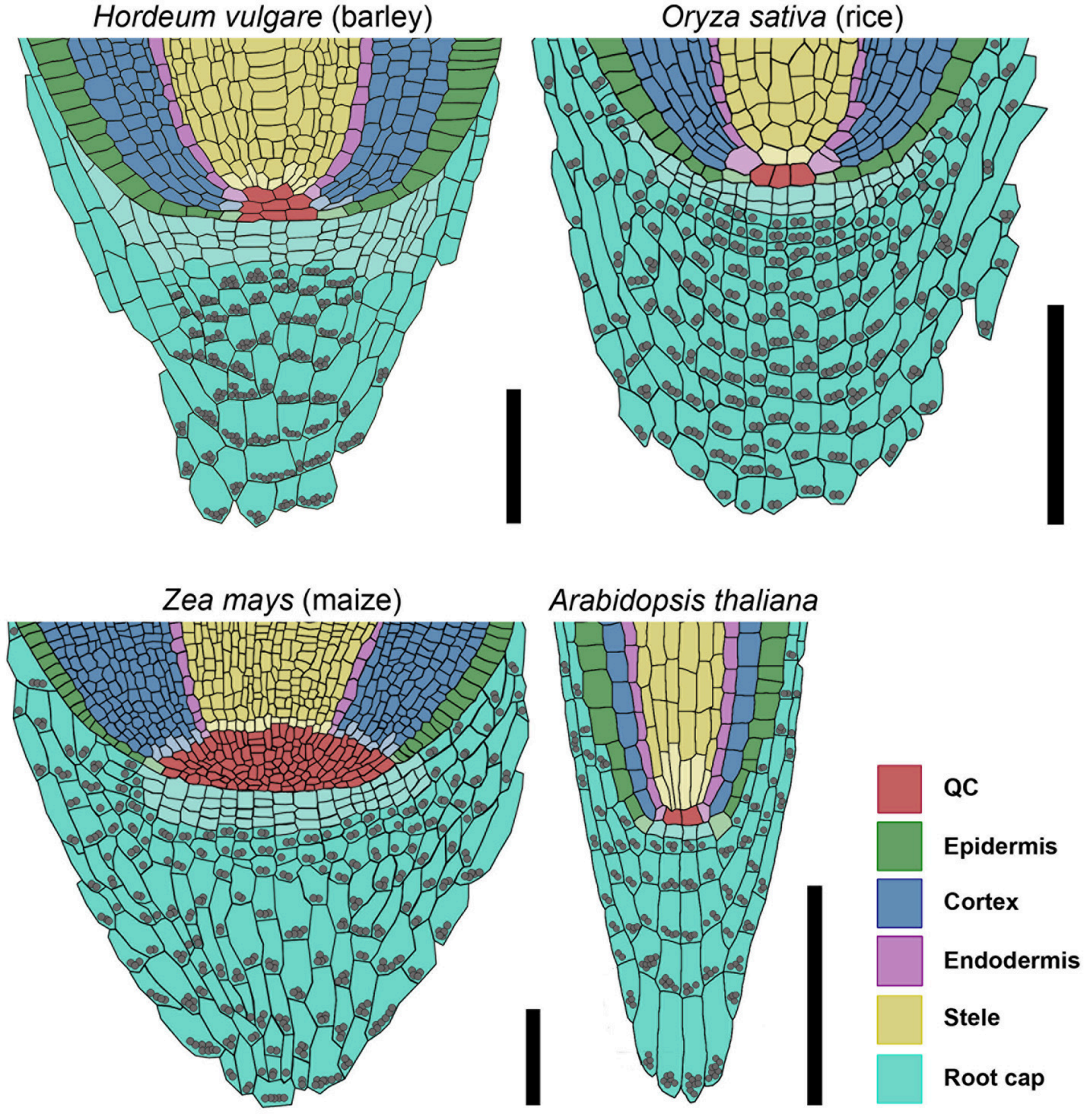
Models of barley, rice, maize, and *Arabidopsis* root stem cell niches. Cell types are marked by color code according to the legend, stem cells that give rise to different tissues are depicted in the respective light colors; gray spheres represent starch granules in the root; the rice stem cell niche was created according to Ni et al. (2014) and Wang et al. (2014); the maize stem cell niche was created according to Kerk and Feldman (1994), Jiang and Feldman (2005) and Jiang et al. (2010); scale bar 100 μm.

To understand the fundamental concepts of root growth, it is important to compare the differences in operating mechanisms between higher plants from the two major groups, monocots and dicots. Here, primary root growth is enabled by root meristems at the tip of the roots, which harbor the stem cell niche that provides the precursors for the various root tissues. Root stem cell niches of different plants share a similar architecture, but the sizes of the stem cell niches, the number of stem cells, the origin of differentiating root cell types and the signaling systems that control cell fates differ significantly. While the principle frameworks governing root meristem functions in the model dicot *Arabidopsis* have been established and are basically understood, far less is known about root meristem functions in monocot species. Analysis of grass root meristems has focussed on maize and rice, and to a lesser extent on Brachypodium (Hardtke and Pacheco-Villalobos, 2015), which all represent examples of closed meristems with discrete initials that, in most cases, give rise to individual cell files. The stem cell niches differ vastly in size between these species, with 4–6 QC cells in rice, but 800–1,200 QC cells in maize (Figure 11; Jiang et al., 2003; Ni et al., 2014). For rice, the origins of cortex and endodermis have been studied in more detail. Here, a stem cell abutting the QC gives rise to several cortex layers and the endodermis via a series of ordered anticlinal and periclinal divisions, resembling the scenario in *Arabidopsis*, with 4 QC cells and a shared initial giving rise to a single cortex cell layer and the endodermis (Dolan et al., 1993; Ni et al., 2014). Similarly, a shared initial generates the lateral root cap and the epidermal cell file, while a single layer of stem cells distal to the QC forms the columella in *Arabidopsis* (Dolan et al., 1993). The overall architecture combined with clonal analysis revealed that lateral root caps and epidermis can be traced back to different stem cells in rice and maize (Hochholdinger et al., 2004b; Wang et al., 2014). Our overall understanding on gene functions regulating the root stem cell niches in monocot species is still very limited, and mostly based on comparative analysis with *Arabidopsis*. Here, we have analyzed for the first time the root meristem architecture of *H. vulgare* (barley), as one of the most important crop species, with the aim to identify commonalities and characteristic features of monocot stem cell systems. We found that, regarding size and general architecture, the barley root meristem occupies an intermediate position between those of maize and rice. Our combined EdU staining and analysis of *HISTONE H4* expression patterns identified approximately 30 slowly dividing cells at the QC position (Figures 4, 5). Unlike in *Arabidopsis* we could not find a clearly defined cell region with complete quiescence, but rather an area displaying a gradual quiescence, with the highest quiescence in the cell layer adjacent to the root cap (Figure 4). This resembles the situation in maize, where quiescence and size of the QC are highly variable. The barley QC is thus considerably larger than that of rice, where each QC cell neighbors dedicated initials (Figure 11; Ni et al., 2014). There, rare asymmetric divisions of QC cells serve to replace adjacent stem cells, while QC cell divisions in barley may also serve to expand the QC size. Although we identified the barley QC based on a lower cell division rate, all QC cells were found to divide within a 24 h time window (Figure 3). This is in stark contrast to the QCs of rice or maize, where no cell divisions in the QC were observed even within a 48 h window (Ni et al., 2014; Jiang et al., 2003). In *Arabidopsis*, less than 20% of all QC cells divided within a 24 h period (Vanstraelen et al., 2009; Cruz-Ramírez et al., 2013). Rarely dividing QC cells have been implicated to be protected from DNA damage and act as a genetic “cache” to replace damaged stem cells. However, for this purpose alone, rather modest differences in cell cycle frequency between QC and surrounding stem cells might be sufficient (Cruz-Ramírez et al., 2013). Importantly, barley generates several seminal roots which might be competing for resource allocation, explaining the wide range of cell divison rates and resulting meristem lengths that we noted earlier (Figures 1B,C). A lower quiescence of the barley QC might thus reflect the physiological state during rapid growth phases. An earlier study by Clowes (1984) found a correlation between the number of cells in the QC and the root diameter, suggesting that bigger roots might need a larger QC, either as a source of DNA-damage protected cells, or as provider of short-range signals for surrounding stem cells (van den Berg et al., 1997; Cruz-Ramírez et al., 2013).

The origin of endodermis and cortex and the regulation of their formation is well researched in *Arabidopsis* and, to a more limited extend, also in rice. In *Arabidopsis*, the cortex cell layer and the endodermis originate from a common initial (Dolan et al., 1993). Because there are more cortex cell layers in rice than in *Arabidopsis* (5 cortex cells layers, one layer of sclerenchyma, and one layer of exodermis, Rebouillat et al., 2009), the sequence of initial divisions is more complex. Rebouillat and colleagues summarize that first an anticlinal cell division near the QC generates the epidermis-endodermis initial, followed by eight successive asymmetric periclinal cell divisions that generate the endodermis, sclerenchyma layer, exodermis and five cortex layers (Rebouillat et al., 2009). A later study confirms this cell division pattern in regard to the endodermis and cortex, but states that the epidermis is not derived from the same stem cell (Ni et al., 2014). For barley, our studies of the cell wall arrangement by mPS-PI staining suggest that the endodermis and a variable number, 1–3, of inner cortex cell layers are derived from a common ancestor, the ICEI, while 2–4 outer cortex cell layers originate from a different precursor (OCI) (Figure 6, Supplementary Movie 1). Notably, formative cell divisions that generated new cortex cell layers occurred at a distance to the QC, indicating that they are either not controlled by the QC itself, or that longer range signals are operating that act over several cell diameters. In *Arabidopsis*, a so-called middle cortex layer is initiated by periclinal cell divisions at a short distance to the QC, which were dependent on the SCARECROW (SCR) transcription factor function, and repressed by gibberellic acid (GA) signaling (Paquette and Benfey, 2005). The independent and distinct origin of inner and outer cortex cells in barley could be reflected in physiological differences between these cell types. In rice, inner and outer cortex cells differ significantly in their cell wall composition and morphology, and in their relative contribution to the ground tissue mass and aerenchyma (Henry et al., 2016). Reporter lines for genes expressed in certain root tissues and marker lines to trace back the cell divisions are not yet available in barley, but would further contribute to increasing our knowledge about the cell lineages in the root.

The barley columella consists of 4 stem cell layers, capped with about 10 layers of differentiated columella cells carrying starch granules, similar to the columella systems of rice or maize (Jiang et al., 2010; Wang et al., 2014). The stem cells in the layer proximal to the QC divide more rapidly than distal ones, indicating that divisional activity is promoted by the QC. In *Arabidopsis*, the columella stem cells are maintained by a CLE40 dependent feedback regulation between QC and differentiated cells (Stahl et al., 2009). We identified 21 genes encoding putative CLE-family peptides in the available barley genome data (Mayer et al., 2012). Alignments with CLE peptide sequences from rice and *Arabidopsis* resulted in the identification of one predicted peptide with the same amino acid sequence in the CLE motif as FCP1. In rice and barley, treatment with the FCP1 or HvCLE402 peptide induced premature differentiation of the proximal root meristem, similar to the observations made for *Arabidopsis* roots (Figure 9). However, in contrast to *Arabidopsis*, the barley distal root meristem displayed no differentiation of columella stem cells (Figure 9). Interestingly, the HvCLE402 peptide triggered differentiation of *Arabidopsis* columella stem cells (Figure 10), which suggests that the receptors perceiving the CLE40 peptides from different species are closely related, but that the mechanisms maintaining distal stem cell populations in barley act independently of the CLE40-dosage.

We have here provided a first framework for a more detailed analysis of root development and stem cell niches in the major crop plant barley. We uncovered commonalities with other monocot species, but also significant functional differences that highlight the importance of a comparative approach in plant developmental studies.

## Author contributions

GK, YS, and RS conceived the project, GK, YS, MV, and RS planned the experiments, GK performed all experiments, GK and RS wrote the manuscript, all authors contributed to the final version.

### Conflict of interest statement

The authors declare that the research was conducted in the absence of any commercial or financial relationships that could be construed as a potential conflict of interest.
