# Supplementary material for: Unique and Conserved Features of the Barley Root Meristem
Source: Front Plant Sci. 2017 Jul 21;8:1240. doi: 10.3389/fpls.2017.01240 (PMC5519606; doi:10.3389/fpls.2017.01240)
Supplement: Supplementary file 2 [file DataSheet1.DOC]

Supplementary Material

# Unique and conserved features of the barley root meristem

Gwendolyn K. Kirschner^1^, Yvonne Stahl^1^, Maria von Korff-Schmising^2^, Rüdiger Simon^1^ *

^1^ Institute for Developmental Genetics, Heinrich Heine University, Düsseldorf, Germany

^2^ Institute for Plant Genetics, Heinrich Heine University Düsseldorf, c/o Department of Plant Developmental Biology, Max Planck Institute for Plant Breeding Research, Cologne, Germany

*** Correspondence:**Rüdiger Simon
Ruediger.simon@hhu.de

# Supplementary Figures and Tables

## Supplementary Figures

**
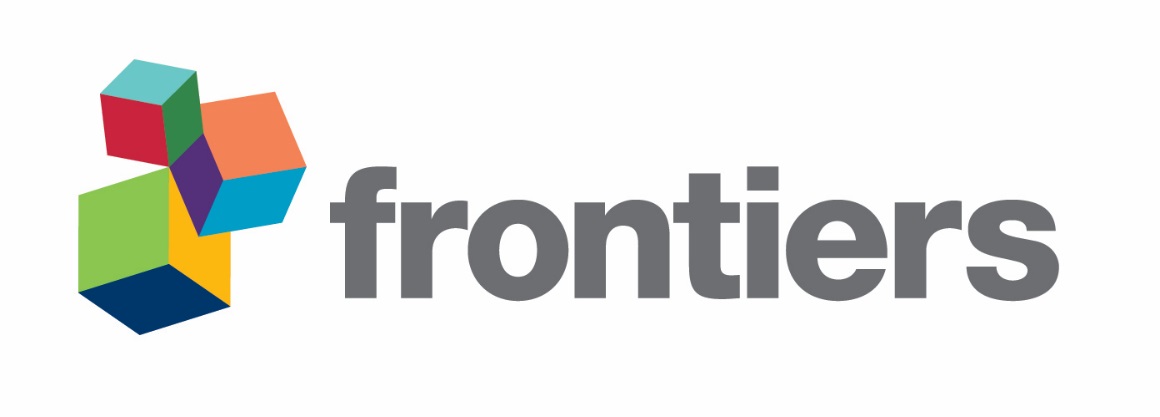
**

**Supplementary Movie 1.** **Cell divisions patterns of the cortex and endodermis initials in the root stem cell niche**

Z-stack through the barley stem cell niche; images were acquired at a spacing of 0.5 µm as indicated in the upper left; watershed segmentation was used to distinguish between individual cells, which are marked by different colours; white asterisks mark the ICEI, black asterisks mark the OCI; red frame marks the QC region; ICEI and OCI are visible in the center of the root, which is marked by the appearance of the metaxylem; abbreviations according to Figure 6 A.
